# Risk communication formats for low probability events: an exploratory study of patient preferences

**DOI:** 10.1186/1472-6947-8-14

**Published:** 2008-04-10

**Authors:** James G Dolan, Stephen Iadarola

**Affiliations:** 1 Department of Medicine, University of Rochester and Unity Health System, 1555 Long Pond Rd, Rochester, New York, USA

## Abstract

**Background:**

Clear communication about the possible outcomes of proposed medical interventions is an integral part of medical care. Despite its importance, there have been few studies comparing different formats for presenting probabilistic information to patients, especially when small probabilities are involved. The purpose of this study was to explore the potential usefulness of several new small-risk graphic communication formats.

**Methods:**

Information about the likelihoods of cancer and cancer prevention associated with two hypothetical cancer screening programs were used to create an augmented bar chart, an augmented grouped icon display, a flow chart, and three paired combinations of these formats. In the study scenario, the baseline risk of cancer was 53 per 1,000 (5.3%). The risk associated with cancer screening option A was 38 per 1,000 (3.8%) and the risk associated with screening option B was 29 per 1,000 (2.9%). Both the augmented bar chart and the augmented grouped icon display contained magnified views of the differences in cancer risk and cancer prevention associated with the screening programs. A convenience sample of 29 subjects (mean age 56.4 years; 76% men) used the Analytic Hierarchy Process (AHP) to indicate their relative preferences for the six formats using 15 sequential paired comparisons.

**Results:**

The most preferred format was the combined augmented bar chart + flow diagram (mean preference score 0.43) followed by the combined augmented icon + augmented bar chart format (mean preference score 0.22). The overall differences among the six formats were statistically significant: Kruskal-Wallis Chi Square = 141.4, p < 0.0001. The three combined formats all had statistically significant higher preferences scores than the single format displays (p < 0.05).

**Conclusion:**

These findings suggest that patients may prefer combined, rather than single, graphic risk presentation formats and that augmented bar charts and icon displays may be useful for conveying comparative information about small risks to clinical decision makers. Further research to confirm and extend these findings is warranted.

## Background

Clear communication about the possible risks and benefits of proposed medical interventions has been an integral part of medical care since the doctrine of informed consent was adopted. In recent years, it has become increasingly more important due to changes in the accepted model of the doctor-patient relationship that promote more active involvement of patients in decisions about their care, the rise of evidence-based medical practice, and the increasing emphasis placed on preventive measures that are intended to reduce future health risks [1,2]. It is likely to become more important in the future as new information about personal health risks becomes available through work in the "new sciences" such as medical genomics, metabolic profiling, and proteomics [3]. Richard Smith, former editor of the British Medical Journal, recently called risk communication the "main work of doctors" [4].

Current recommendations for communicating information about uncertain future events emphasize the importance of presenting data in a balanced manner that avoids framing effects, provides baseline risk information, and uses graphic risk displays whenever possible. Recommended risk displays include bar charts, grouped icon displays (also known as dot or face diagrams), and flow diagrams [5-7]. The use of multiple risk presentation formats has also been suggested [7,8].

Despite the importance of risk communication, there have been few empirical studies comparing different graphic formats for presenting probabilistic information to patients for use in shared decision-making [8-12]. In comparative studies, both bar charts and icon displays have been more effective than simple numeric statements and several other graphic formats for comparing the outcomes of alternative decision options [13,14]. Ordered icon displays have also been shown to minimize decision biases due to vivid anecdotal information and to be well understood by patients over 75 years old [16,17]. Flow diagrams, developed by Gigerenzer and colleagues, have been shown to help people better understand risk forecasts as well as more complex probabilistic data, such as the change in risk of disease after a positive or negative test result [18,19].

A limitation of the currently available data is that most studies to date have compared risk displays using relatively common events with likelihoods of 20% or more. Outcomes with expected likelihoods of less than 5% occur frequently in medical decision making situations and in many cases have an important impact on medical decisions. Probably the most common of these low likelihood outcomes are risks of serious adverse events. For example, in 2004 the drug rofecoxib, a COX-2 inhibitor, was removed from the market in the United States due to concerns about an increase in the annual rate of myocardial infarctions from one per thousand to four per thousand, an absolute risk increase of three per thousand [15].

Small risks are difficult to depict in bar charts and icon displays because it is hard to include all of the necessary information in a single display. Studies with both bar charts and icon displays have shown that graphs that do not include information about the chances of both experiencing and not experiencing the outcome can lead to misinterpretation of the data presented [12,16]. The need to properly illustrate this part-to-whole relationship is what makes it difficult to use these formats to convey information about low likelihood events

A possible solution to this problem is to use graphs that include a special section designed to highlight information about low likelihood events. This approach has been used successfully in two studies that elicited probability estimates from patients using graphs that included a "magnified" zone for probabilities less than 1% [22,23]. Whether this approach can also be used to create effective graphic displays for conveying low likelihood information to clinical decision makers is currently unknown.

This study was designed as the first step in a series of investigations designed to address the question of how to most effectively present information about low likelihood events to patients and other clinical decision makers. The purpose of this study was to explore the potential usefulness of several new small-risk graphic communication formats.

## Methods

### Study population

The study population consisted of a convenience sample of middle-aged to elderly adults, recruited through word of mouth and distribution of informational materials, who volunteered to participate in a study of medical decision making. Patients were recruited from three separate sites in Rochester NY: a general maintenance shop, a weekly social gathering at an elderly retirement community, and a medical research institute contained within the University of Rochester. No patients had prior knowledge of the project or the study hypotheses. Formal health status was not assessed. However, subjects at the maintenance shop and research institute were working regularly at the time of the study and the subjects at the retirement community were well enough to attend a weekly social function. This study was a pilot study performed in connection with a larger project that was approved by the University of Rochester Research Subjects Review Board. The data were collected anonymously. All subjects gave consent to participate.

### Study intervention

The study intervention was a computer-assisted comparison of six different graphic communication formats for displaying the differences in effectiveness of two hypothetical cancer screening programs. In the study scenario, the baseline risk of cancer was 53 per 1,000 (5.3%). The risk associated with cancer screening option A was 38 per 1,000 (3.8%) and the risk associated with screening option B was 29 per 1,000 (2.9%). These absolute risks and risk reductions are comparable to outcomes seen with currently recommended colorectal cancer screening tests [17]. Although screening by itself does not reduce cancer risk, we did not include details about screening-related treatments to simplify the problem and focus attention on the graphic displays.

**Figure 1:**
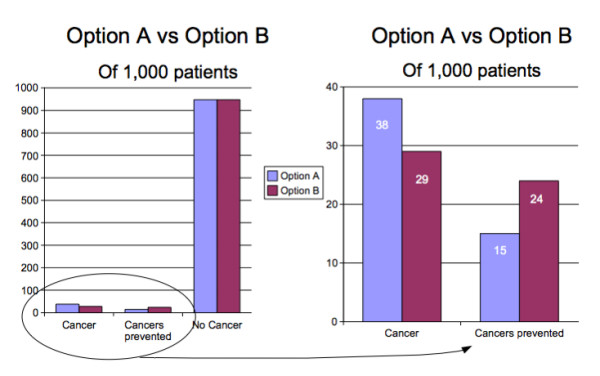
**The augmented bar chart**. The left hand panel is a standard bar chart showing the entire dataset. The right hand panel magnifies the differences between the two options so the magnitude of the differences can be seen more clearly.

**Figure 2:**
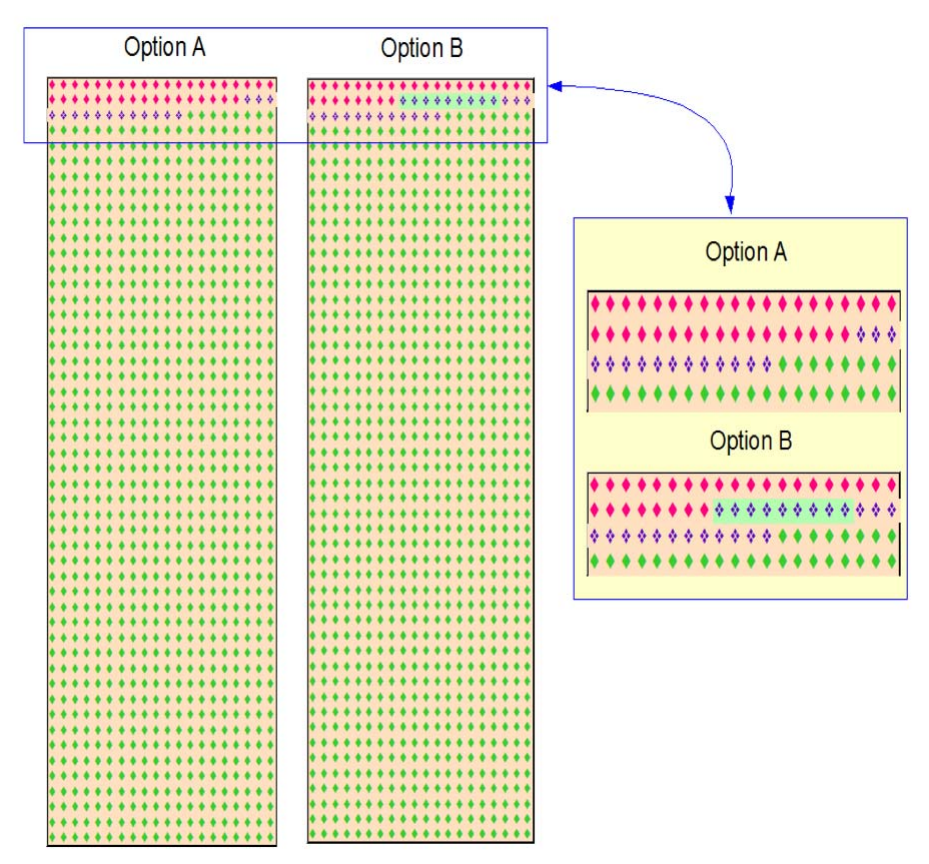
**The augmented icon display**. The left hand panel is a standard icon display showing the entire dataset. The right hand panel magnifies the differences between the two options so the magnitude of the differences can be seen more clearly. The red diamonds indicate patients with cancer, the green diamonds indicate patients without cancer, and the broken diamond symbol () indicates cancers prevented through screening and screening-related interventions.

**Figure 3:**
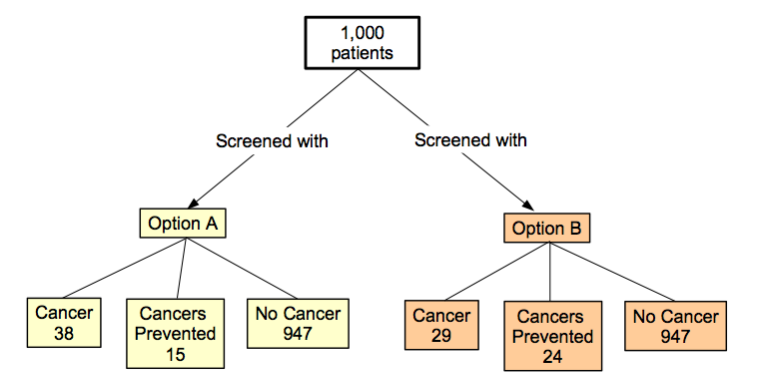
The flow diagram.

Three of the formats studied were based on currently recommended graphic displays: a flow diagram, a vertical bar chart, and a grouped icon display. The bar chart and icon display were augmented to include both an overall depiction of the possible outcomes associated with each screening option and a magnified view of the differences between them in cancer risk and likelihood of cancer prevention. The resulting diagrams are shown in Figures 1, 2, 3.

**Figure 4:**
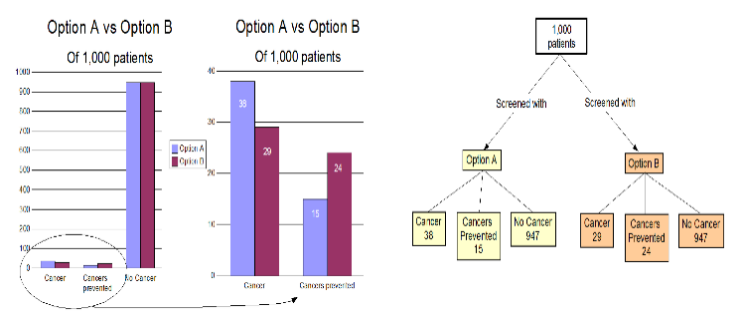
**Example paired display format**. This figure shows the combined format consisting of both an augmented bar chart and a flow diagram.

The other three formats included in the study were paired combinations of the three single formats: a flow diagram plus an augmented icon display, a flow diagram plus an augmented bar chart, and an augmented icon display plus an augmented bar chart. An example of a combined format is shown in Figure 4.

**Figure 5:**
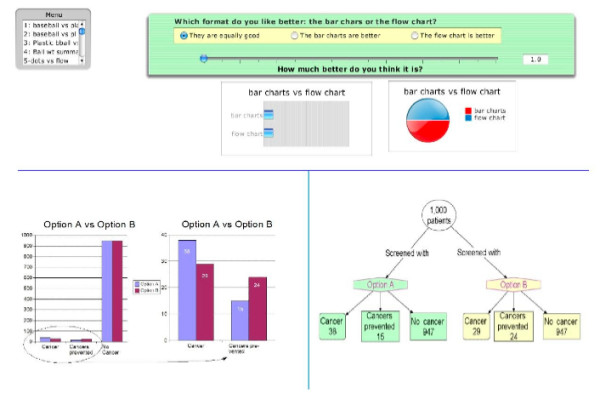
**Example preference comparison screenshot**. This figure shows the screen used by the study subjects to make the comparisons among the risk presentation formats. The slider used to indicate their strength of preference, if any, is shown in the top panel. The magnitude of preference was indicated in the numeric box to the right and in the linked horizontal bar charts and pie chart below. The panel in the upper left is the menu screen used to move from one comparison to the next.

After a brief explanation of the study and a training exercise that involved judging the relative weights of three balls, the subjects were shown two of the six diagrams on a computer screen and asked to indicate if they liked both formats equally or if they preferred one to the other for comparing the effectiveness of the two screening options for preventing cancer. If one was preferred, they were asked to indicate their strength of preference using a dynamic slider that showed a range of preferences from equal to nine times more preferable. The relationships between different settings on the slider and relative strengths of preference were illustrated by linked horizontal bar and pie charts. An example of the screen used to make these comparisons is shown in Figure 5. This process was repeated until all six formats were compared, a total of 15 comparisons. Participants needed approximately 45 minutes to one hour to complete the study. The computer program used to perform these assessments is attached: see Additional file 1.

Quantitative preference scores summarizing the comparisons among the formats were calculated using the Analytic Hierarchy Process (AHP). To create the AHP-based numeric preference scores, each subject's comparisons were entered into a matrix and the normalized right eigenvector of the matrix was calculated. The resulting eigenvector values define the formats' preference scores. Because the eigenvector is normalized, the scores range from 0 to 1 and the sum of all scores equals 1. Scores are interpreted as indicating each format's relative strength of preference compared to the others, measured on a ratio scale.

The reliability of each subject's comparisons was assessed by calculating the consistency ratio, a standard measure of the reliability of the pairwise comparisons used to generate the preference scale that is routinely used in AHP applications [22]. Consistency ratios run from 0 to 1, with 0 indicating a perfectly consistent set of comparisons. Traditionally, consistency ratios ≤ 0.10 have been considered acceptable; in practical applications this cut-off is often increased to ≤ 0.20 [25]. For this study the latter cutoff was used and defined *a priori*.

### Statistical analysis

We analyzed the data to see if there were significant differences among the six presentation formats in subject preferences. Because the response data were not normally distributed, we tested this hypothesis using Kruskal-Wallis analysis of variance. The level of statistical significance was set *a priori *as an alpha level of 0.05. Differences between individual formats were tested using the Bonferroni multiple comparison test to maintain an alpha of 0.05. All statistical analyses were done using NCSS 2000 [26].

## Results

**Figure 6:**
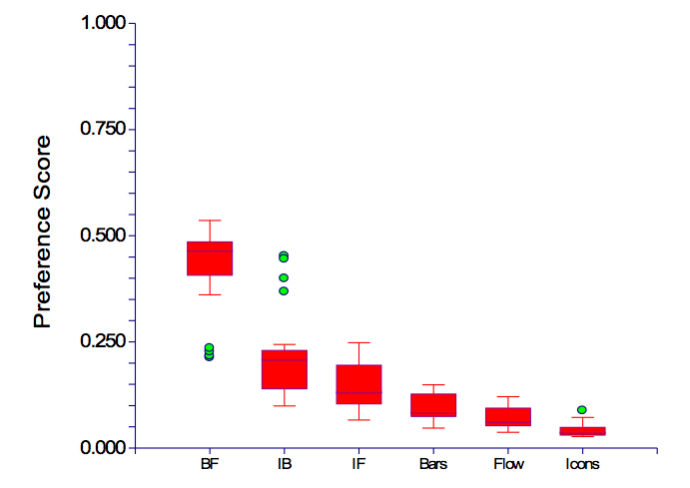
**Box plot showing the preferences scores for the six risk presentation formats**. Abbreviations: BF = augmented bar chart + flow diagram; IB = augmented icon display + augmented bar chart; IF = augmented icon display + flow chart; Bars = augmented bar chart; Flow = flow diagram; Icons = augmented icon display. Box plot details: The horizontal line inside the box indicates the median value. The inner box indicates the inter-quartile range that runs between the 25^th ^to 75^th ^percentiles. The upper line extending from the box indicates the largest value between the 75^th ^percentile and the point that is 1.5 times the inter-quartile range. The lower line extending from the box indicates the smallest point between the 25^th ^percentile and 1.5 times the inter-quartile range. The circles represent values that lie outside these ranges.

The mean age of the study population was 56.4 years. There were 22 men and 7 women. The preference scores for the six presentation formats are summarized in the box plot shown in Figure 6. The most preferred format was the combined augmented bar chart + flow diagram with a mean preference score 0.43. This format is shown in Figure 4. The combined augmented icon display + bar chart was second most preferred with a mean preference score of 0.22. A small group of patients strongly preferred this format as indicated by the outlier circles on the box plot. The combined augmented icon display + flow chart had the third highest mean preference score, 0.15. The three single presentation formats were the least preferred with mean preference scores of 0.10 for the augmented bar chart, 0.07 for the flow diagram, and 0.04 for the augmented icon display.

The overall differences in preferences among the six formats were statistically significant: Kruskal-Wallis Chi Square = 141.4, p < 0.0001. The Bonferroni multiple comparison test indicated that the mean preference scores for all three combined formats were significantly different from all other options and the mean preference score for the augmented bar chart was significantly different from the mean score of the augmented icon display, (p < 0.05).

The consistency indices for the comparisons ranged from 0.08 to 0.20 with a mean of 0.135. All subjects met the predefined standard for acceptable consistency of 0.20 or less.

## Discussion

A recently issued set of guidelines for creating patient decision aids recommends the use of multiple risk presentation formats [7]. These results support this recommendation. The most preferred format was a combined format and all three combined formats were more preferred than the three single format options included in the study.

We were unable to find previous studies that have assessed patient preferences for single versus combined graphic risk presentation formats. In a study that compared text formats for presenting treatment effectiveness information, Sheridan and colleagues found that patient understanding was worse for a combined format than several single formats [18]. The discrepancy between this result and ours may reflect differences in the way people process textual versus graphical information [19,20]. Alternatively, it could be due to the differences in the outcomes studied: preferences versus understanding.

Given the limited scope of this study, we do not know why the patients preferred the combined formats so strongly. The cost-benefit theory of decision strategy choice posits that the way information is displayed affects a person's choice of how they will process the information based on anticipated effort and anticipated accuracy [19]. It is possible that the increased variety of information presentation provided by the combined formats led the patients to perceive that they could be used to make more accurate decisions with less effort than the single format displays. Additional research is warranted to confirm our results and to address additional outcomes including understanding of the information presented, cognitive effort, the rationale underlying risk presentation preferences, and the effect of presentation preferences on the decision making process.

This study also demonstrates the usefulness of using the Analytic Hierarchy Process as a technique for creating measurement scales in situations where no pre-existing scale exists. Although the AHP is most widely known as a multi-criteria decision making method, it is fundamentally a system of measurement that derives a ratio-level scale from a series of pairwise judgments [21-28]. In addition to producing a robust measurement scale, the AHP also yields an assessment of the consistency of the component judgments that can be used as an indicator of the reliability of the measurement process. The AHP therefore is a valuable assessment tool that can enhance our ability to investigate preferences and other important, but difficult to measure, concepts.

This study has several limitations. First, the only outcome studied was patient preference. At least two studies have shown that the most preferred risk formats are not necessarily the same as the most effective formats for risk communication in medical decision making [13,14]. Assessing preferences as the initial step in evaluating new risk communication formats, however, has the advantage of providing easy to obtain information about an important dimension of a risk display that can be used to help plan additional investigations. Second, only two likelihoods for a single outcome were studied. We were, therefore, not able to determine if there is an interaction between the magnitude of the risk being presented and preferred display format. Third, the study population was a small volunteer convenience sample of people who were not facing a real decision. Fourth, the reproducibility of preferences over time was not tested. Finally, only one version of the newly created graphs was studied. Whether patient preferences are affected by different color schemes, axis formatting, the size of the display, and other design characteristics is unknown.

## Conclusion

Despite these limitations, the results of this study suggest that patients may prefer combined, rather than single, graphic risk presentation formats and that augmented bar charts and icon displays may be useful for conveying comparative information about small risks to clinical decision makers. Further research to confirm and extend these findings is warranted.

## Competing interests

The author(s) declare that they have no competing interests.

## Authors' contributions

JD conceived and designed the study, performed the data analysis, and drafted the manuscript. SI created the data collection computer interface, recruited study subjects, administered the study interviews and compiled the results. Both authors read and approved the final manuscript.

## Pre-publication history

The pre-publication history for this paper can be accessed here:



## Supplementary Material

Additional file 1 Risk communication survey.swf. The instrument used to obtain the preferences from study subjects. Click here for file
